# Indocyanine-enhanced mouse model of bleomycin-induced lung fibrosis with hallmarks of progressive emphysema

**DOI:** 10.1152/ajplung.00180.2022

**Published:** 2023-01-10

**Authors:** Andrea Grandi, Erica Ferrini, Laura Mecozzi, Roberta Ciccimarra, Matteo Zoboli, Ludovica Leo, Zahra Khalajzeyqami, Alex Kleinjan, Clemens W. G. M. Löwik, Gaetano Donofrio, Gino Villetti, Franco Fabio Stellari

**Affiliations:** ^1^Chiesi Farmaceutici S.p.A., Corporate Pre-Clinical R&D, Parma, Italy; ^2^Department of Veterinary Science, University of Parma, Parma, Italy; ^3^Department of Medicine and Surgery, University of Parma, Parma, Italy; ^4^Department of Veterinary Medical Sciences, University of Bologna, Bologna, Italy; ^5^Department of Pulmonary Medicine, Erasmus University Medical Center, Rotterdam, The Netherlands; ^6^Department of Radiology and Nuclear Medicine, Erasmus University Medical Center, Rotterdam, The Netherlands

**Keywords:** bleomycin, emphysema-like, fluorescent and micro-CT imaging, indocyanine green, mouse model of pulmonary fibrosis

## Abstract

The development of new drugs for idiopathic pulmonary fibrosis strongly relies on preclinical experimentation, which requires the continuous improvement of animal models and integration with in vivo imaging data. Here, we investigated the lung distribution of bleomycin (BLM) associated with the indocyanine green (ICG) dye by fluorescence imaging. A long-lasting lung retention (up to 21 days) was observed upon oropharyngeal aspiration (OA) of either ICG or BLM + ICG, with significantly more severe pulmonary fibrosis, accompanied by the progressive appearance of emphysema-like features, uniquely associated with the latter combination. More severe and persistent lung fibrosis, together with a progressive air space enlargement uniquely associated with the BLM + ICG group, was confirmed by longitudinal micro-computed tomography (CT) and histological analyses. Multiple inflammation and fibrosis biomarkers were found to be increased in the bronchoalveolar lavage fluid of BLM- and BLM + ICG-treated animals, but with a clear trend toward a much stronger increase in the latter group. Similarly, in vitro assays performed on macrophage and epithelial cell lines revealed a significantly more marked cytotoxicity in the case of BLM + ICG-treated mice. Also unique to this group was the synergistic upregulation of apoptotic markers both in lung sections and cell lines. Although the exact mechanism underlying the more intense lung fibrosis phenotype with emphysema-like features induced by BLM + ICG remains to be elucidated, we believe that this combination treatment, whose overall effects more closely resemble the human disease, represents a valuable alternative model for studying fibrosis development and for the identification of new antifibrotic compounds.

## Graphical abstract

**Figure:**
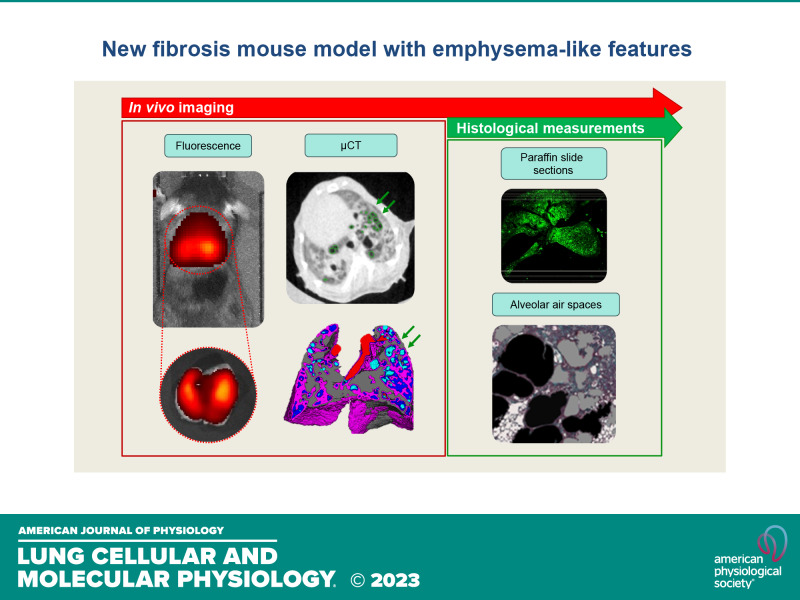

## INTRODUCTION

Idiopathic pulmonary fibrosis (IPF) is a heterogeneous, chronic disease characterized by progressive lung scarring and impaired pulmonary function, ultimately leading to respiratory failure and death. A truly resolving pharmacological therapy has yet to be identified, and lung transplantation often remains the only treatment option (1, 2). Even though animal models of IPF are crucial tools to address this urgent unmet medical need, none of the currently available models fully replicates the human disease condition (3, 4). Among the animal, especially mouse, models of IPF, administration of bleomycin (BLM), an antibiotic chemotherapeutic agent with marked pulmonary toxicity associated with inflammatory overproduction of multiple cytokines and reactive oxygen species (ROS) ultimately leading to extensive DNA damage and cell death, is the most widely used (5).

However, this model does not faithfully reproduce all the main features of the human disease and some drawbacks, such as the spontaneous resolution of fibrosis and a significant degree of interanimal variability, have been reported (6, 7). Even though BLM administration by oropharyngeal aspiration (OA) has been shown to result in more homogeneous parenchymal alterations compared with intratracheal delivery (8), variations in the amounts of BLM reaching the lungs may still cause significant confounding effects. To try to manage this variability and achieve a stratification of BLM-treated mice based on different levels of in vivo determined lung damage, we resorted to indocyanine green (ICG), a fluorescent tracer widely used for different diagnostic, clinical, and preclinical applications (9–11). Importantly, except for ROS and heat production upon photothermal treatment (12) and necrotic lesions with dysfunctional mitochondrial activity only observed in a very few specific cell types, such as retinal pigment epithelium (13), no significant cytotoxic effects have been reported for ICG since its Food and Drug Administration approval in the 1950s.

We thus mixed ICG with BLM to monitor lung distribution and exposure following OA in mice through an in vivo imaging system (IVIS). Surprisingly, and in stark contrast with the very short half-life commonly observed with intravenously administered ICG (14), we found that OA-administered ICG was retained in the lungs for up to 21 days. Moreover, although ICG alone did not cause any detectable lung parenchymal alteration, a single OA administration of BLM + ICG triggered severe pulmonary fibrosis with distinctive emphysema-like features that were not observed upon a double BLM instillation without the ICG dye. We followed up on this finding with micro-computed tomography (CT) in vivo time-course analysis of disease progression, comparing the effects of single compounds (BLM and ICG) with those of their combination (BLM + ICG) in individual mice longitudinally. To further investigate the effects observed in vivo, we performed immunofluorescence assays and subsequent quantification of collagen I, an extracellular matrix component, fibroblast activation protein 1 alpha (FAP-1α), and the apoptotic marker caspase-3, on formalin-fixed paraffin-embedded (FFPE) sections of murine lungs. We then tested ICG, BLM, and BLM + ICG on murine macrophages (RAW 264.7) and on epithelial (LA-4) cell lines in vitro. Quantitative lung fibrosis parameters derived from micro-CT scans, expressed as normo-, hypo-, non-aerated, or hyper-inflated lung tissues (15), were then compared with the results of histological analyses, bronchoalveolar lavage fluid (BALF) leukocytes and cytokines profiling at different time points (7, 14 and 21 days) after compound administration.

## MATERIALS AND METHODS

### In Vivo Methods

#### Experimental animals.

Female inbred C57Bl/6j (8 wk old, 20 ± 1 g of body weight) mice were purchased from Envigo, Italy (San Pietro al Natisone, Udine, Italy). Before use, animals were acclimatized for 7–10 days to the local vivarium conditions (room temperature: 20°C–24°C; relative humidity: 40%–70%; 12-h light-dark cycle), having free access to regular rodent chow and softened tap water. Sterile sunflower seeds and hydrogel were supplied as diet integration to prevent excessive body weight loss.

Animals were housed five per cage under standard conditions at our animal facility, in compliance with the procedures and principles outlined in the European Directive 2010/63 UE, Italian D.Lgs 26/2014 and the revised “Guide for the Care and Use of Laboratory Animals” (National Research Council Committee, US, 2011). All animal procedures were conducted in an AAALAC (Association for Assessment and Accreditation for Laboratory Animal Care) certified facility at Chiesi Farmaceutici and were authorized by the Italian Ministry of Health with protocol number 809/2020-PR and by the internal AWB (Animal Welfare Body).

A visual analog scale (0–10) for pain assessment was assessed daily by a designated veterinarian or trained technicians. VAS ≥6 and/or body weight loss ≥ 20% were considered as humane endpoints, as well as signs of dyspnea or apathy evaluated by a designated veterinarian.

#### Oropharyngeal administration.

The solutions instilled via OA were prepared as follows:

Bleomycin hydrochloride (Baxter Oncology GmbH, Germany) was diluted in 0.9% NaCl at a working concentration of 0.5 mg/mL (BLM); indocyanine green (ICG, Sigma-Aldrich, St. Louis, MO) was dissolved in sterile water and diluted with saline (Sal) at a working concentration of 2.5 mg/mL (Sal + ICG). The solution was prepared in a vial covered with aluminum foil to prevent light exposure and was used immediately after solubilization preparation to avoid compound precipitation; BLM and ICG were dissolved at 1 mg/mL and 5 mg/mL concentrations, respectively, and then mixed (1:1 volume ratio) to obtain the final BLM + ICG solution. Sal and BLM were administered twice (indicated as “TW”), at *days 0* and *4*, whereas Sal + ICG and BLM + ICG were administered once, at *day 0* (Supplemental Fig. S1, *A* and *B*).

For each solution, 50 µL was administered to mice as described previously (8, 16); briefly, animals were placed on the intubation platform, hanging by their incisors over a wire, then the tongue was gently pulled out with forceps, using a small laryngoscope and with a micropipette, the liquid was placed onto the distal part of the oropharynx while the nostrils were closed.

Three independent experiments were carried out. As follows, the total number of mice used per group: Sal (*n* = 15) and Sal + ICG (*n* = 15), BLM (*n* = 15) and BLM + ICG (*n* = 36).

#### In vivo and ex vivo fluorescence imaging.

Animals were lightly anesthetized with 2% isoflurane, shaved, and imaged using IVIS Lumina II (PerkinElmer Inc., Waltham, MA) (17).

ICG (λ_Ex_ = 790 nm, λ_Em_ = 840 nm) fluorescent signal was quantified in calibrated (radiant efficiency) units [(photons/s/cm^2^/str)/(µW/cm^2^)] in Sal + ICG and BLM + ICG mice using the software Living Image version 4.3.1. (PerkinElmer Inc., Waltham, MA). Mice were imaged in a prone position at 0, 7, 14, and 21 days after OA treatment; an average of total fluorescence signal emitted from the chest region was calculated for each mouse. Two representative Sal and BLM mice were also imaged as negative controls. At each time point, subgroups of mice were euthanized, and lungs were collected for histological analysis. Before formalin fixation, lungs were imaged for ex vivo fluorescence detection as already described. Thereafter, two paraffin-embedded lung slides from Sal + ICG and BLM + ICG samples were cut with 5-µm thickness and fluorescence at 800 nm detected by Odyssey (LI-COR Biosciences - GmbH).

#### Micro-CT: acquisition protocol and image postprocessing analysis.

Micro-computed tomography (micro-CT) was performed at *days 7, 14*, and *21* using a Quantum GX Micro-CT (PerkinElmer, Inc. Waltham, MA). Mice were lightly anesthetized with 2% isoflurane and images were acquired with the following parameters: 90 KV, 88 µA, and total scan time of 4 min (over a total angle of 360°). The “high speed” acquisition protocol was used, and a respiratory-gated technique was applied. The entire set of projection radiographs was reconstructed using a filtered back-projection algorithm with a Ram-Lak filter and resulted in two stacks of 512 slices with a nominal resolution of 50 µm.

The reconstructed datasets were analyzed with Analyze software (Analyze 12.0; Copyright 1986–2017, Biomedical Imaging Resource, Mayo Clinic, Rochester, MN). Following the analysis protocol, CT scans were filtered and then converted from gray levels to CT numbers (Hounsfield Units, HU).

For the quantitative assessment of the aeration degrees, the total lung volume was extracted from the reconstructed image (9, 15) through manual segmentation. The lung aeration compartments were then determined by applying “HU preclinical ranges” (15) dividing the whole parenchyma in normo-aerated ([–860, −435] HU), hypo-aerated ([−435, −121] HU), non-aerated ([−121, +121] HU), and hyperinflated areas ([−1,040, −860] HU), expressed as percentage of total lung volume.

The hypo- and non-aerated lung tissue areas refer to those with a low gas/tissue ratio, which was previously developed to quantify lung fibrosis progression and evaluate antifibrotic drug efficacy (15).

#### Bronchoalveolar lavage, cells count, and cytokines.

At 7, 14, and 21 days, after the in vivo imaging, subsets of 5 (Sal), 5 (BLM), 5 (Sal + ICG), and 12 (BLM + ICG) mice were euthanized with an overdose of anesthetic followed by bleeding from the abdominal aorta. Bronchoalveolar lavage fluid (BALF) was then harvested by gently washing the lungs with 0.6-mL sterile Hank’s balanced salt solution (HBSS) containing 1 mM ethylenediaminetetraacetic acid (EDTA) and 100 mM 4-(2-hydroxy-ethyl)-1-piperazineethansulphonic acid (HEPES); the collected fluid was centrifuged at 300 *g* for 10 min at 4°C. The cell pellet was resuspended in 0.2 mL of phosphate buffer solution (PBS), and leukocytes and cell populations were determined using an automated cell counter (Dasit XT 1800 J, Sysmex), while supernatants were frozen at −80°C. At a later time, BAL supernatants were thawed, and the levels of multiple cytokines and chemokines were determined, using a Magnetic Luminex Assay (R&D Systems, Minneapolis, MN), according to the manufacturer’s instructions; neutrophil elastase and total TGF-β1 levels were determined with the SimpleStep ELISA Kit (Abcam) and the LEGEND MAX Total TGF-β1 ELISA Kit (Biolegend), respectively.

#### Histological assessment of the lung.

After BAL collection, the lungs were removed and inflated with a cannula through the trachea by gentle infusion of 0.6 mL of 10% neutral-buffered formalin and fixed for 24 h. For histological assessment, the samples were dehydrated in a graded ethanol series, clarified in xylene, and paraffin-embedded. Sections of 5-μm thickness were cut with a rotary microtome (Slee Cut 6062; Slee Medical, Mainz, Germany) and then stained with hematoxylin and eosin (H&E) and Masson’s trichrome (TM), according to the manufacturer’s specifications (Histo-Line Laboratories). The whole slide images (WSIs) were acquired by the NanoZoomer S-60 Digital slide scanner (Hamamatsu, Japan) for analysis. Three sections for each lung sample were stained with TM and scored on a scale of 0–8 by two independent investigators who were blinded to the treatments. Fibrotic modifications were assessed morphologically and semiquantitatively graded according to the scale defined by Ashcroft et al. (18) and modified by Hübner et al. (19) (see Supplemental Fig. S2). The final score was expressed as a mean of individual scores observed across all microscopic fields. To quantify the frequency distribution of pulmonary fibrosis, the Ashcroft scores were graded in three classes of increasing values ranging from 0 to 3 (mild), 4 (moderate), and ≥5 (severe) and expressed as a percentage (20).

#### Immunofluorescence assays.

Paraffin-embedded sections (4-μm thickness) were dewaxed and processed as described previously (21). Sections were stained with an anti-collagen I antibody (2.5 μg/mL; ab88147; Abcam), an anti-FAP-1-α rabbit polyclonal antibody (13.5 μg/mL; ab53066; Abcam), and an anti-cleaved caspase-3 antibody (diluted 1:50; D17S-9661S; Cell Signaling). Secondary antibodies (Alexa 647-anti mouse IgG3-115-605-209; Alexa 488-anti rabbit IgG-111–545-144, and Rhodamine Red-X-anti rabbit IgG 711-295-209; all from Jackson Immunoresearch) were diluted 1:500 before utilization. Sections were incubated overnight with primary antibodies, which were then revealed by fluorochrome-tagged secondary antibodies; for negative control, the primary antibody was omitted, or alternatively, tissues were incubated with mouse IgG3 isotype control (2.5 μg/mL; ab183954; Abcam) and with rabbit IgG isotype control (13.5 μg/mL; ab37415; Abcam). Nuclei were counterstained with DAPI and sections were mounted with fluorescent mounting medium. Stained slides were scanned and saved as digital images using a multichannel fluorescence acquisition instrument (Nano-Zoomer S60, Hamamatsu, Japan).

Digital slide images (NDPITools) were uploaded, recorded, and analyzed with QuPath (version 0.2.3), an open-source software for image analysis (22). DAPI staining was used for cell segmentation with optimized parameters to better identify cell nuclei. Once all images were aligned, autofluorescence was subtracted from FITC and TRITC channels (21). Biomarkers’ levels were measured within segmented cell regions and data were expressed as a percentage of positive cells. Data were normalized on total cells number.

#### Histomorphometric assessment of airspace enlargements.

Alveolar air spaces enlargement and alveolar destruction, in accordance with emphysema definition (23), were histomorphometrically evaluated by the parameters of mean linear intercept (MLI) and alveolar airspace area (AAA) (24–29). The alveolar air spaces area was dimensionally categorized for the analysis as normal (0–100 µm), medium (101–300 µm), large (≥ 300 µm), labeled in white, gray, and black, respectively. The distribution of AAA during the time course was expressed as a percentage of air content normalized to the total lung parenchyma.

#### Digital analysis (mean linear intercept and alveolar airspace area).

The mean linear intercept (MLI) and alveolar airspace area (AAA) were semiautomatically detected, through morphological and color-thresholding tools, using an ImageJ plug-in (https://med.nyu.edu/nolanlab) for the MLI quantification and Visiopharm’s Quantitative Digital Pathology software (Visiopharm, Hørsholm, Denmark) for the AAA.

MLI provides the mean free distance between gas exchange surface in the acinar airway complex (23). MLI was measured within three regions of interest (ROI = 0.811 mm2) through the semiautomatic ImageJ plug-in (23). The data, expressed in pixels, are converted into micron and the horizontal and vertical mean value provides the MLI for each ROI. The alveolar airspace area was obtained using Visiopharm software through computer applications developed in-house. Through comprehensive color recognition, Visiopharm provides a range of automated multivariate classifiers that use several image-learning methods. Based on TM whole slide images, the workflow to determine the AAA was divided into four sequential APPs: *1*) image borders detection, *2*) identification of histological structures, *3*) airways size categorization, and *4*) airspace size metrics.

*1*) Image borders detection: The purpose of this step is to determine the working area available for quantitative analysis. A combination of three “image classes” was used for training the decision forest classifier, an ensemble learning method that takes advantage of constructing a multitude of classifiers, to recognize the image principal pixels. The postprocessing step in combination with “morphological tools” (e.g., erode, dilate, open, close, fill hole) allows this application to define the lung profile, surrounding the tissue and excluding the outside.

*2*) Identification of histological structures: Decision Forest algorithm was trained by an experienced operator to recognize blood vessels, airways, and tissue (all remaining structures) found in the histological image stained with TM. The identification of blood vessels and airways from the whole section was possible, particularly, following a postprocessing list with a specific order.

*3*) Airways size categorization: Focusing only on airways, with the help of “morphological tools” designed on Visiopharm to highlight different sizes, it was possible to proceed with a dimensional categorization of the “alveolar air spaces”: normal (0–100 μm), medium (101–300 μm), and large (≥ 300 μm).

*4*) Airspace size metrics: The last application, working on labeled region, returns quantitative data generated by algorithms with “calculation options.” This was applied to the airways categories, allowing definition of the percentage occupied by each of three categories of total lung structure.

### In Vitro Methods

#### Cell culture.

Murine macrophage (RAW 264.7, ATCC Cat. No. TIB-71, RRID:CVCL_0493) and epithelial (LA-4, ATCC Cat. No. CCL-196, RRID:CVCL_3535) cell lines were purchased from ATCC and grown in T-75 flasks (Corning) until they reached the 80% of confluence. For subculture, they were detached from plate using 0.025% Trypsin-EDTA solution (Gibco, Thermo Fisher Scientific) and centrifuged for 4 min at 125 *g* at room temperature. LA-4 were cultured in F-12K nutrient mixture (Gibco, Thermo Fisher Scientific) + 1X penicillin/streptomycin (Gibco, Thermo Fisher Scientific) + 15% FBS (Gibco, Thermo Fisher Scientific), whereas RAW 264.7 were cultured in DMEM (Gibco, Thermo Fisher Scientific) + 1X penicillin/streptomycin/glutamine (Gibco, Thermo Fisher Scientific) + 10% FBS. A subcultivation ratio of 1:3 was generally applied in T-75 flasks every 2–3 days and confluence was carefully evaluated with an inverted microscope (×10 magnification). The passage range of 5–10 was considered for in vitro experiments. Experiments were repeated at least three times with similar results.

#### Cell treatments.

After 24 h from seeding, cells were starved for 3 h using the same culture medium supplemented with 2% FBS. 2X solutions of ICG and BLM were freshly prepared in culture medium to achieve final concentrations of 0.01 and 0.1 mg/mL for ICG, as reported in previous studies (30–32), and 3 and 50 µg/mL for BLM. Following starvation, the medium was replaced, and cells were treated with ICG and BLM alone, by adding an equal volume of medium to the 2X solutions of the two compounds, and in combination for 4 and 24 h. Untreated cells were used as controls.

#### Fluorescence microscopy.

LA-4 were seeded on glass coverslips (Sigma-Aldrich) positioned inside 12 multiwell plates. After treatment, cells were fixed with paraformaldehyde 4% and stained with 4′,6-diamidino-2-phenylindole, dihydrochloride (DAPI, Thermo Fisher Scientific) for 5 min. After three washings with PBS, the cytoplasmatic or nuclear ICG uptake (red) was evidenced with fluorescence microscope Nikon Eclipse Ti2 (Nikon) and cell nuclei were highlighted in blue.

#### Cell viability assay.

Cells were seeded (5 × 10^4^ cells/well) in 0.5 mL of complete medium in 48 multiwell plates. After treatment, the supernatants were replaced with 0.5 mg/mL thiazolyl blue tetrazolium bromide (MTT solution, Sigma-Aldrich) diluted in serum-free culture medium. Plates were then incubated for 2 h at 37°C. The deposition of violet crystals in the bottom well reveals the presence of metabolic active and vital cells. The solution was then removed, and crystals were dissolved in 0.2 mL of dimethyl sulfoxide (DMSO, Sigma-Aldrich). Absorbance was immediately quantified using a plate reader (xMark Microplate Spectrophotometer, Bio-Rad) at 570 nm wavelength and optical densities (O.D.) were quantified through the Microplate Manager Software 6 version 6.3 (Bio-Rad). At each time point of observation, the viability was measured in triplicate for both the cell lines, and biological replicates were performed at least twice with similar results. Results were expressed as % of vital cells compared with untreated cells as control (considered as 100% by default) at each time point.

#### Real-time RT-qPCR.

After 4 h and/or 24 h treatment, RAW 264.7 and LA-4 cells were washed with Hank’s balanced salt solution (HBSS), and RNA was extracted using the RNeasy Plus Mini kit (Qiagen) following the instructions protocol. The concentration of RNA was measured using the NanoDrop 2000 (Thermo Fisher Scientific) and retrotranscribed using SuperScript VILO Master Mix (Invitrogen). TaqMan Assays Mm00432322_m1 (*CASP7*), Mm01204974_m1 (*FAS*), and Mm00725412_s1 (*ACTA2*) were used to measure the expression of *CASP7*, *FAS*, and *ACTA2* genes, using *GAPDH* as housekeeping gene. RT-qPCR was performed with QuantStudio 7 Flex Real-Time PCR System (Thermo Fisher Scientific). Cycle threshold (Ct) for each gene and sample was recorded and 2-ΔΔCt analysis was conducted using untreated RAW 264.7 or LA-4 as controls (fold change corresponding to 1).

### Statistics

Statistical analyses were performed using Prism 8 software (GraphPad Software Inc., San Diego, CA). All data are presented as mean ± SEM. One- or two-way analysis of variance (ANOVA) was performed, followed by Sidak’s or Tukey’s multiple comparisons post hoc tests. Normal distribution was assessed through Shapiro–Wilk test accompanied by visual inspection of QQ-plots. Sample size was calculated with a priori Power Analysis (G*Power Version 3.1.2) considering Ashcroft Score as endpoint. For all the applied tests, a *P* < 0.05 (*) was considered as statistically significant.

## RESULTS

### Prolonged Lung Retention of ICG Revealed by In Vivo and Ex Vivo Fluorescence Imaging

**Figure 1.**
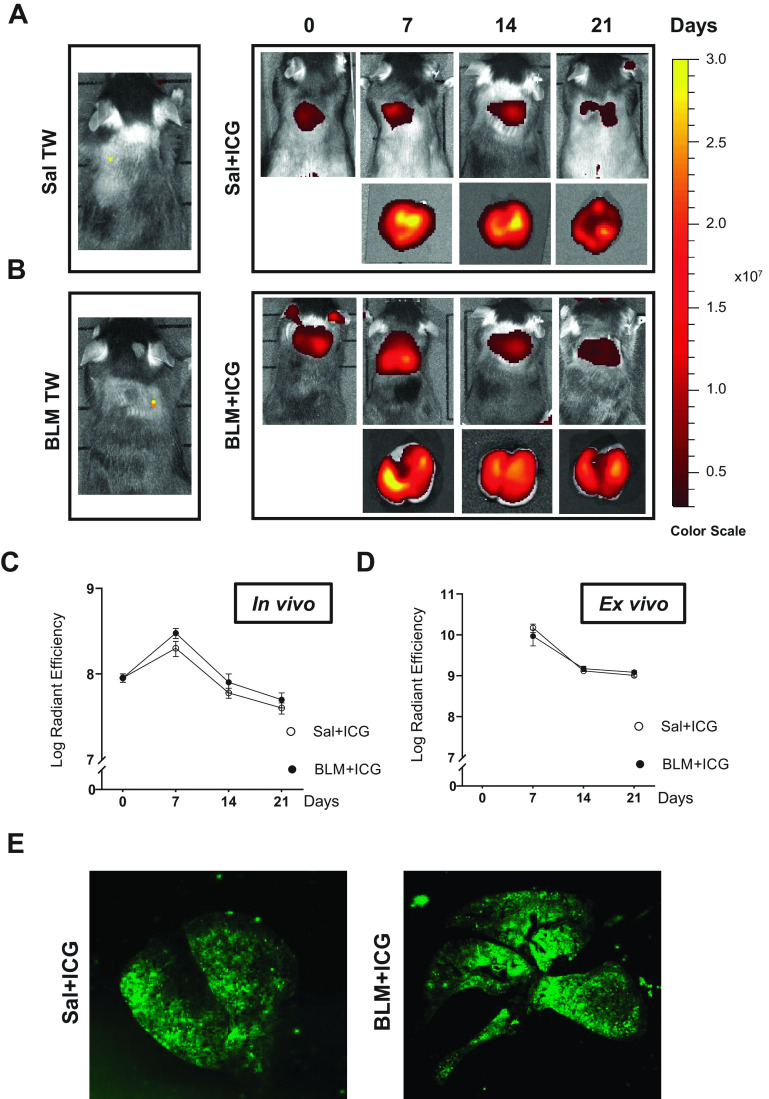
In vivo and ex vivo fluorescence imaging. *A*: representative in vivo and ex vivo fluorescence images of Sal- and Sal+ICG-treated mice at 0, 7, 14, and 21 days after OA, as indicated. *B*: same as *A* for the BLM- and BLM+ICG-treated groups. *C*: time course of the in vivo quantified fluorescence signals. *D*: same as *C* for ex vivo quantified fluorescence signals. *E*: representative images acquired by near-infrared analysis (Odyssey scanner, LI-COR; see materials and methods for details) of paraffin-embedded lung slides from Sal+ICG- and BLM+ICG-treated animals. Data are given as means ± SE of three biological replicates. Numbers of animals (*n*) were as follows: Sal, Sal+ICG, and BLM groups, *n* = 5 for each time point; BLM+ICG groups, *n* = 12 for each time point. BLM, bleomycin; ICG, indocyanine green; OA, oropharyngeal aspiration; Sal, saline.

We initially traced lung accumulation and distribution of OA-administered ICG, alone and in combination with BLM, by in vivo fluorescence analysis. Representative in vivo fluorescence images of Sal, BLM, Sal + ICG, and BLM + ICG mice in prone position and ex vivo lung-associated signals at different time points are shown in Fig. 1, *A* and *B*, respectively. As revealed by these images, a well-detectable fluorescence signal localized to the chest region was observed in all mice exposed to ICG, both alone and in combination with BLM. No statistically significant difference in the measured fluorescence signal could be detected at any time, both in vivo and ex vivo, between Sal + ICG- and BLM + ICG-treated animals (Fig. 1, *C* and *D*); as expected, no such signal was detected in Sal- nor BLM-treated mice.

The ICG signal was detectable immediately after OA and throughout a series of predefined time points. It peaked at *day 7* and gradually decreased thereafter, both in vivo and ex vivo. At *day 21*, a sizeable ICG signal could still be detected (7 and 9 log radiant efficiency in vivo and ex vivo, respectively; Fig. 1, *C* and *D*). This signal could also be detected by near-infrared fluorescence visualization performed with an Odyssey scanner on histological slides derived from Sal + ICG- and BLM + ICG-treated animals after 21 days (Fig. 1*E*).

The above results indicate a durable and unexpectedly prolonged persistence in the lungs of both ICG alone as well as in association with bleomycin.

### Micro-CT Analysis

**Figure 2.**
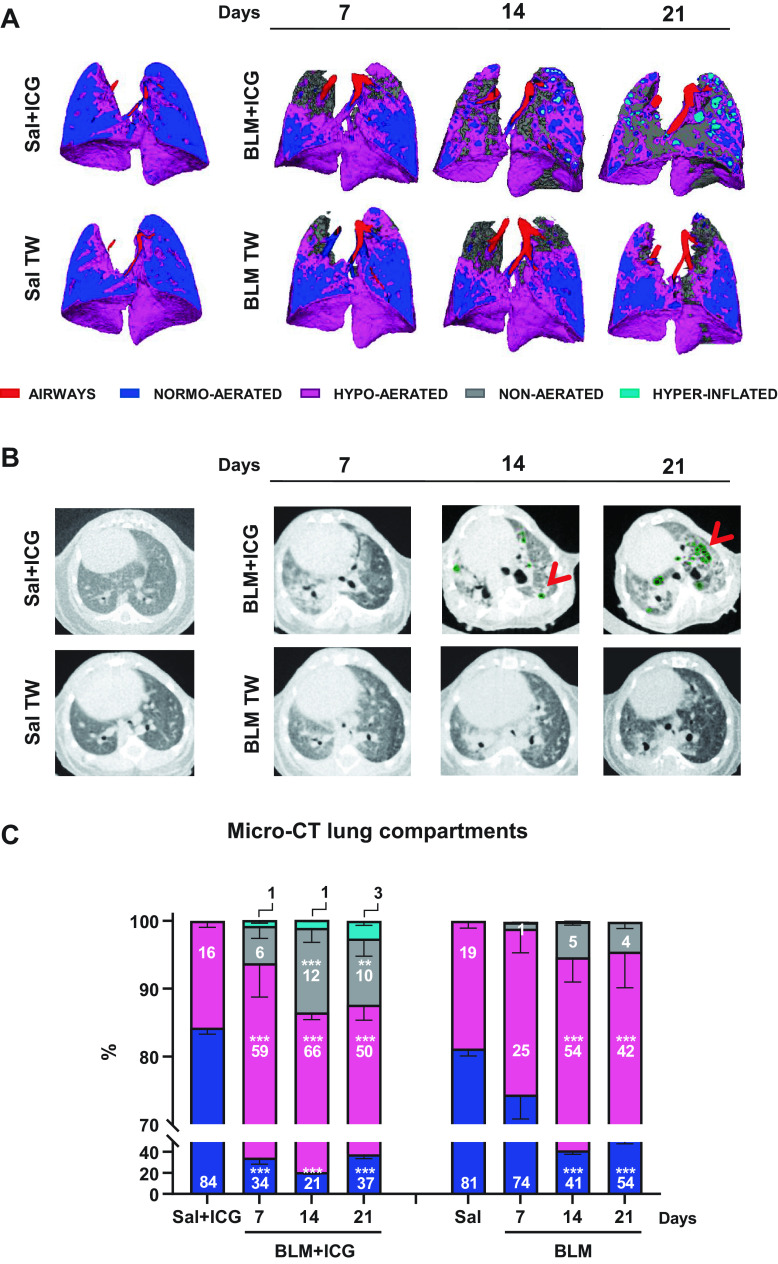
Different degrees of lung aeration assessed by micro-CT. *A*: representative three-dimensional lung renderings of Sal, BLM, Sal+ICG, and BLM+ICG mice at 7, 14, and 21 days; the color codes for the airways (trachea and bronchus) and for the different types of observed lung tissue alterations are shown at the bottom. *B*: representative coronal sections of micro-CT images of Sal, BLM, Sal+ICG, and BLM+ICG mice at 7, 14, and 21 days. Red arrows and green dots indicate areas of emphysema. *C*: percentages of normo-, hypo-, non-aerated, and hyperinflated tissues as derived from micro-CT analyses performed on Sal, BLM, Sal+ICG, and BLM+ICG mice at 7, 14, and 21 days; the color codes for the different types of lung tissue alterations are the same as in *A*. The values for the Sal and Sal+ICG groups (not shown) were constant throughout all time points. Two-way ANOVA with Sidak’s correction for multiple comparisons was applied to the following numbers of animals (*n*): Sal, Sal+ICG, and BLM *n* = 5 for each time point; BLM+ICG *n* = 12 for each time point; ***P < 0.001 BLM+ICG vs. Sal+ICG and BLM vs. Sal at the indicated time points for all compartments. Data (means ± SE) were derived from three independent experiments. BLM, bleomycin; CT, computed tomography; ICG, indocyanine green; OA, oropharyngeal aspiration; Sal, saline.

Micro-CT analysis was used, next, to dissect the macro-morphological features of the lung alterations induced by BLM and ICG, both alone and in combination. As revealed by the representative three-dimensional (3-D) lung segmentations in Fig. 2*A*, which were obtained by micro-CT imaging performed at different time points, a progressive appearance of hypo-aerated and non-aerated lung tissue (i.e., dense areas with low gas/tissue ratios) was observed upon BLM or BLM + ICG instillation but not after treatment with Sal + ICG, which was indistinguishable from the saline-only control. Interestingly, however, hyperinflated areas (i.e., air-entrapping regions; shown in light blue in Fig. 2*A*) were only detectable at both 14 and 21 days in BLM + ICG-treated mice. A similarly compromised lung parenchyma, with multiple air-filled and bubble-like shaped low-density regions characterized by well-defined margins, progressively accumulating as a function of increasing posttreatment time, was revealed by coronal micro-CT imaging of BLM + ICG but not BLM-only treated mice (Fig. 2*B*).

Normo-, hypo-, non-aerated, and hyperinflated lung areas, as revealed by micro-CT analysis, were thus considered hallmarks of lung compartments with distinctive gas/tissue ratios. Although hypo- and non-aerated lung parenchyma is diagnostic of different degrees of fibrosis, alveolar air space enlargement as seen in hyperinflated areas represents the micro-CT imaging correlate of an emphysema-like phenotype.

Accordingly, individual lung compartments of animals belonging to the BLM-only and the BLM + ICG groups were quantitatively imaged at *days 7, 14*, and *21*, using Sal- and Sal + ICG-treated mice as controls, respectively. As shown in Fig. 2*C*, BLM + ICG mice displayed a significant increase in hypo-aerated (*P* < 0.001 vs. Sal + ICG) and non-aerated (*P* < 0.01 vs. Sal + ICG) areas at all the investigated time points, with a peak for both types of abnormal compartments at *day 14*. A similar increase in underaerated areas was observed at *day 14* in BLM-treated animals but it reached statistical significance (*P* < 0.001 vs. Sal) only for hypo-aerated tissue. In fact, the fractional occurrence of non-aerated tissue, which is indicative of more severe fibrotic lesions, was 5 ± 1% at *day 14* in the BLM group but more than twice as much (12 ± 2%) in the case of the BLM + ICG group.

Hyperinflated tissue areas containing bubble-like shaped structures were not detectable in BLM-treated mice nor in the Sal + ICG or Sal controls, but they were clearly visible in the BLM + ICG group and time-dependently increased at *days 7, 14*, and *21*.

### Histological Assessment of Lung Fibrosis

**Figure 3.**
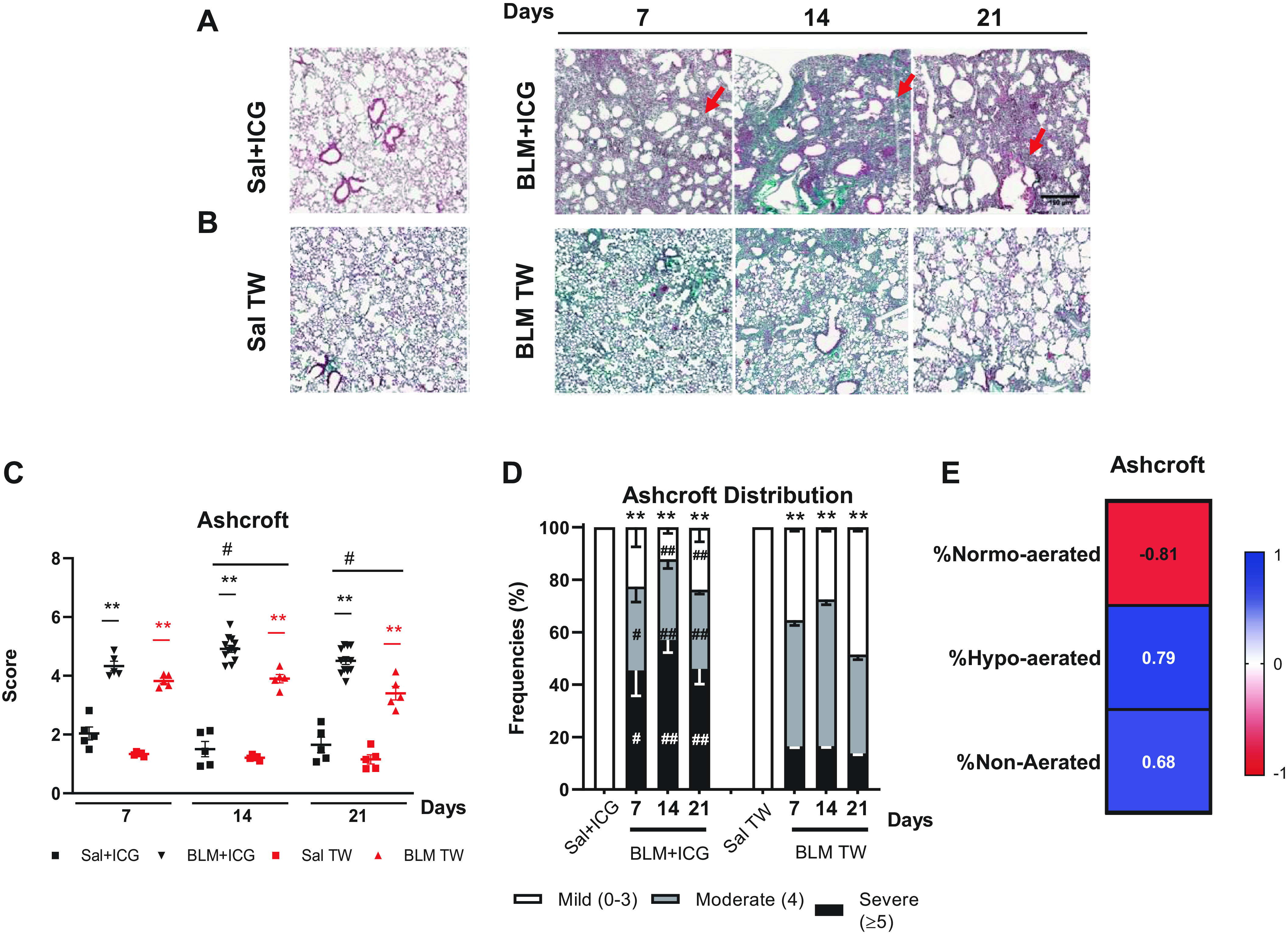
Histological assessment of lung fibrosis. *A*: representative images (×5 magnification; scale bar 150 µm) of Masson’s trichrome-stained lung sections from mice treated with Sal+ICG and BLM+ICG at the indicated times post-OA. *B*: same as *A* for the Sal and BLM-only groups. *C*: time course of Ashcroft score variation in mice treated with Sal+ICG, BLM+ICG, Sal, and BLM. *D*: frequency distribution of the Ashcroft score values in the BLM+ICG and BLM-only groups at the indicated time points; the Ashcroft score frequency distribution for the Sal+ICG and Sal control groups remained essentially constant throughout all time points. *E*: Spearman correlation coefficients for the Ashcroft score compared with %normo-, hypo-, or non-aerated tissue. Two-way ANOVA with Sidak’s correction for multiple comparisons was applied to the following number of animals (*n*): Sal, Sal+ICG, and BLM groups, *n* = 5 for each time point; BLM+ICG groups, *n* = 12 for each time point. **P < 0.01: BLM+ICG vs. Sal+ICG and BLM vs. Sal; #*P* < 0.05 and ##*P* < 0.01 for the BLM+ICG vs. BLM comparisons. Data are the means ± SE of three independent biological replicates. BLM, bleomycin; ICG, indocyanine green; OA, oropharyngeal aspiration; Sal, saline.

Histological analysis was performed on subsets of Sal + ICG and BLM + ICG animals euthanized at *days 7, 14*, or *21* and on parallel tissue samples derived from Sal- and BLM-treated mice (Fig. 3).

Severe lung fibrosis, with the presence of multiple fibroproliferative foci and massive collagen deposition, was observed in BLM + ICG-treated animals. As shown in Fig. 3*A*, it was already visible on *day 7*, worsened on *day 14*, and remained essentially unchanged till *day 21* (Fig. 3*A*). By comparison, BLM-only mice featured a moderate lung parenchymal alteration with very few fibroproliferative foci at *day 7*, followed by the appearance of confluent, substitutive collagen conglomerates at *days 14* and *21*, with partial lung obliteration due to fibrotic proliferation (Fig. 3*B*).

Alveolar walls collapse and destruction, with a marked enlargement of alveolar airspaces, was already visible in lung tissue samples from BLM + ICG animals at *day 7* and further evolved at *days 14* and *21*. This was accompanied by a progressive widening of the balloon-like airspace closely resembling a typical emphysema-like morphology (Fig. 3*A*). No such lung architectural alteration was observed in the BLM, Sal, and Sal + ICG groups (Fig. 3, *A* and *B*). A severe lung fibrosis, significantly higher in the BLM and BLM + ICG groups compared with controls (*P* < 0.01), was revealed by Ashcroft score measurements performed at the different time points, with a clear trend toward further worsening at *day 14* (Fig. 3*C*). Even higher Ashcroft score values were measured in BLM + ICG, compared with BLM-only-treated mice at *days 14* and *21* (*P* < 0.05). Indeed, as shown in Fig. 3*D*, the frequency of severe lesions was significantly higher in the BLM + ICG than in the BLM-only group at *days 7* (*P* < 0.05), *14*, and *21* (*P* < 0.01). Conversely, mild to moderate lesions were markedly lower in the BLM + ICG compared with the BLM-only group (*P* < 0.01), which displayed the highest frequency of moderate rather than severe lesions at all time points. Both BLM groups, however, featured a significantly higher abundance of moderate to severe lesions compared with the control Sal + ICG and Sal groups (*P* < 0.01), which did not differ significantly from each other.

Overall, BLM + ICG-treated mice displayed significantly more severe fibrotic lesions compared with BLM-only animals at all time points, with morphologically distinctive signs of an emphysema-like alteration, most notably, a marked enlargement of the alveolar air space not observed in the latter group. The Ashcroft score of the Sal + ICG, Sal, BLM + ICG, and BLM groups was highly correlated with micro-CT parameters such as normo-, hypo-, and non-aerated areas (Fig. 3*E*).

### Immunofluorescence Quantification

**Figure 4.**
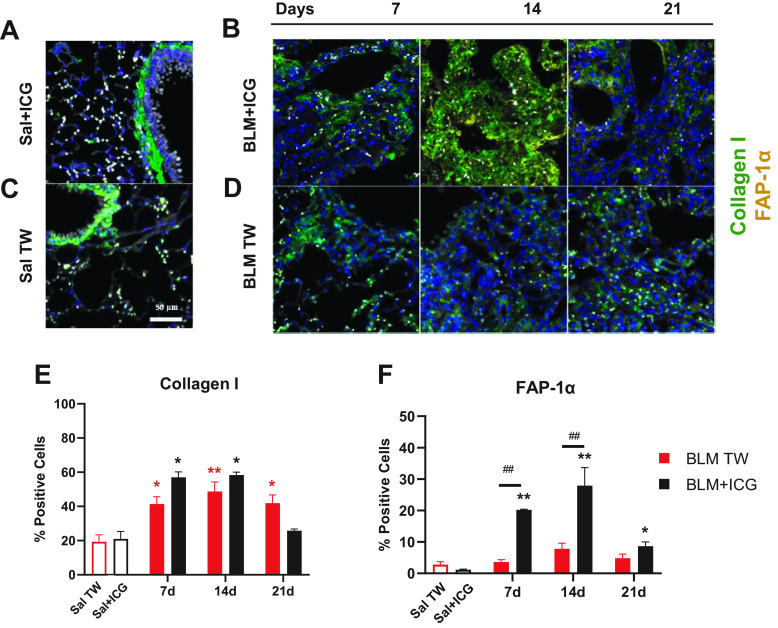
Immunofluorescence staining of Collagen I and FAP-1α. Representative immunofluorescence microphotographs of lung parenchyma (×20 magnification; scale bar 50 µm) from mice treated with Sal+ICG (*A*) and BLM+ICG (*B*) at the indicated post-OA time points. *C* and *D*: same as *A* and *B* for the Sal and BLM-only groups. Parenchymal cells were immunolabeled for Collagen I and FAP-1α; nuclei were visualized with DAPI (blue), Collagen I was stained with Alexa Fluor 647 (green) and FAP-1α with Alexa Fluor 488 (red). *E*: immunofluorescence data quantitation and time-dependent variation of the number of Collagen I-positive cells in mice treated with BLM+ICG or BLM, using Sal+ICG- and Sal-treated animals as controls. *F*: same as *E* for FAP-1α quantitation. Asterisks indicate statistical significance vs. the corresponding control groups (*P < 0.05; **P < 0.01). Hashtags indicate the significance of intergroup (BLM+ICG vs. BLM-only) differences (##*P* < 0.01). BLM, bleomycin; ICG, indocyanine green; OA, oropharyngeal aspiration; Sal, saline.

Immunofluorescence assays were performed on controls (Sal + ICG and Sal), BLM-only-treated, and BLM + ICG mice euthanized at *days 7*, *14*, or *21*. We determined expression levels of Collagen I and FAP-1α, a protein associated with matrix remodeling (Fig. 4, *A*–*D*). A considerable increase of the FAP-1α signal in the BLM + ICG group compared with the BLM-only group was observed, especially on *days 7* and *14* after OA. The Collagen I signal, instead, displayed a similar intensity in the two groups (see Supplemental Fig. S3).

Quantification of the Collagen I signal revealed a peak at *day 14* in both BLM + ICG and BLM-only-treated mice (Fig. 4*E*). Even though no statistically significant difference was observed between the BLM + ICG and BLM-only groups at either of the investigated time points, a significant difference in Collagen I levels was apparent in BLM mice compared with the Saline control group at *day 21*; no significant difference was observed, instead, between BLM + ICG and its control group (Sal + ICG). FAP-1α, the other cellular component we used as a biomarker, is a type II transmembrane protein that is upregulated in activated fibroblasts within areas of active tissue remodeling (33, 34), particularly within fibroblastic foci in IPF. The percentage of FAP-1α-positive cells was significantly higher in BLM + ICG mice compared with the Sal + ICG group at all the investigated time points (*P* < 0.01 *days 7* and *14*; *P* < 0.05 *day 21*), whereas a nonsignificant increase was detected in BLM mice relative to the control Sal group. Moreover, FAP-1α expression levels were much higher in BLM + ICG than in BLM-only mice at *days 7* and *14* (*P* < 0.05, Fig. 4*F*), thus pointing to a much stronger fibroblast activation in the combination treatment group as a sign of a more active tissue remodeling somewhat unique to the BLM + ICG model. An assessment of tissue content distribution and, particularly, the presence of medium and large alveolar spaces were also performed. This analysis allowed to quantify the severity of the presence of balloon-like alterations and the resulting tissue coarctation taking place in the BLM + ICG compared with the BLM-only model. As shown in Supplemental Fig. S4, an increment of the large air space size (>300 µm) in the BLM + ICG group was apparent at all time points and became statistically significant (*P* < 0.05) at *day 21*. This was accompanied by a loss of tissue resulting from coarctation and scarring due to the establishment of emphysematous-like conditions.

### Histomorphometric Evaluation of Air Space Enlargement

**Figure 5.**
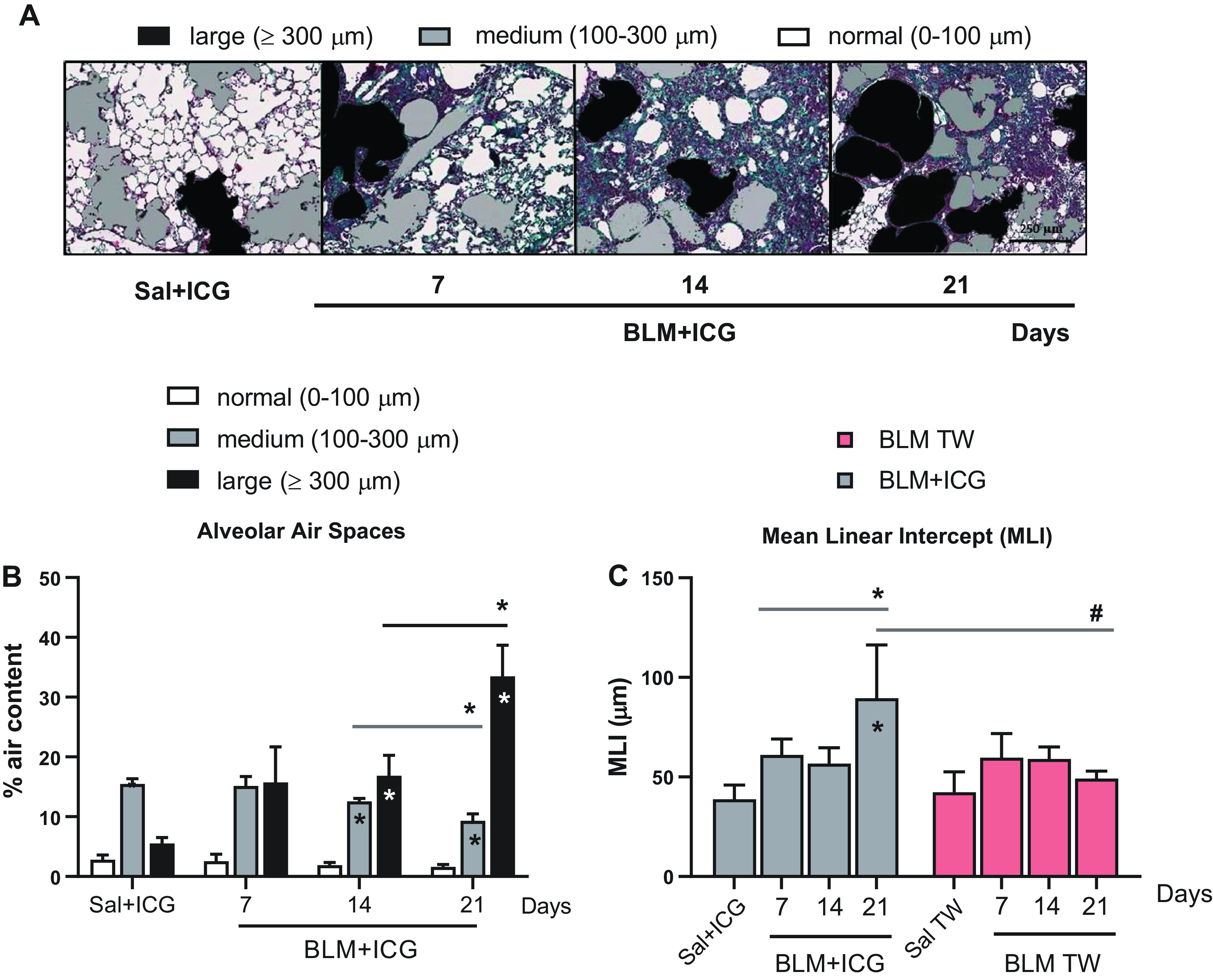
Histomorphometric evaluation of air space enlargement. *A*: representative images of differently sized alveolar air spaces in lung tissue samples from control (Sal+ICG) and BLM+ICG- mice at different times after treatment as indicated (×10 magnification; scale bar 250 µm). Alveolar air space size and mean linear intercept (MLI) for the Sal+ICG and Sal control groups remained essentially constant throughout all time points. Images, shown with original histological colors (Masson’s Trichrome stain), were analyzed with Visiopharm. The air space size categories used for classification are shown at the top. *B*: distribution of the different air space size categories (% air content) in the control (Sal+ICG) and BLM+ICG groups described in *A*. Asterisks within the bars indicate the significance of BLM+ICG vs. Sal+ICG differences; asterisks shown above the bars (horizontal lines) indicate the statistical significance of the differences between the various time points (*P < 0.05). *C*: alveolar air spaces evaluation by mean linear intercept (MLI) analysis of the BLM+ICG and the BLM-only groups at different time points, as indicated. MLI values are compared with the appropriate controls (Sal+ICG and Sal, respectively) within each group and between the two groups (BLM+ICG and BLM-only); asterisks within the bars indicate the significance of the differences with respect to the controls (Sal+ICG or Sal); asterisks shown above the bars (horizontal lines) indicate the statistical significance of the differences between the various time points (*P < 0.05); hashtags indicate the significance of intergroup (BLM+ICG vs. BLM-only) differences (#*P* < 0.05). BLM, bleomycin; ICG, indocyanine green; Sal, saline.

As shown in Fig. 5*A*, in keeping with our previous micro-CT and histological observations, BLM + ICG-treated mice displayed distinctive emphysema-like features with progressive alveolar swelling and air space enlargement. Histological samples from these animals were then used for a more detailed, semiquantitative investigation of these balloon-like alterations. Air space size was classified as normal (0–100 µm), medium (101–300 µm), and large (≥ 300 µm), and the fractional representation (%) of each size category within the total lung parenchyma was quantified at 7, 14, and 21 days after treatment. As shown in Fig. 5*B*, a significantly higher representation of the “large-air space” category, especially at mid/late posttreatment times, was observed in the BLM + ICG group (*P* < 0.05 at *days 14* and *21*) along with a significant decrease of the “medium-size” air space category, compared with the Sal + ICG group.

As revealed by a mean linear intercept analysis performed on the same micrographs (Fig. 5*C*), marked structural abnormalities were indeed apparent in the BLM + ICG group. These peaked at *day 21* and were not detected at any of the examined time points in the Sal + ICG or the BLM-only groups.

The observed changes in MLI, but also in the alveolar airspace area, are once again strongly indicative of an emphysema-like alteration, as initially suggested by micro-CT imaging data, which highlighted the presence of multiple and well-detectable low-density, bubble-shaped structures within the lung parenchyma.

### Effect of the BLM + ICG Combination on BAL Inflammatory and Fibrotic Biomarkers

**Figure 6.**
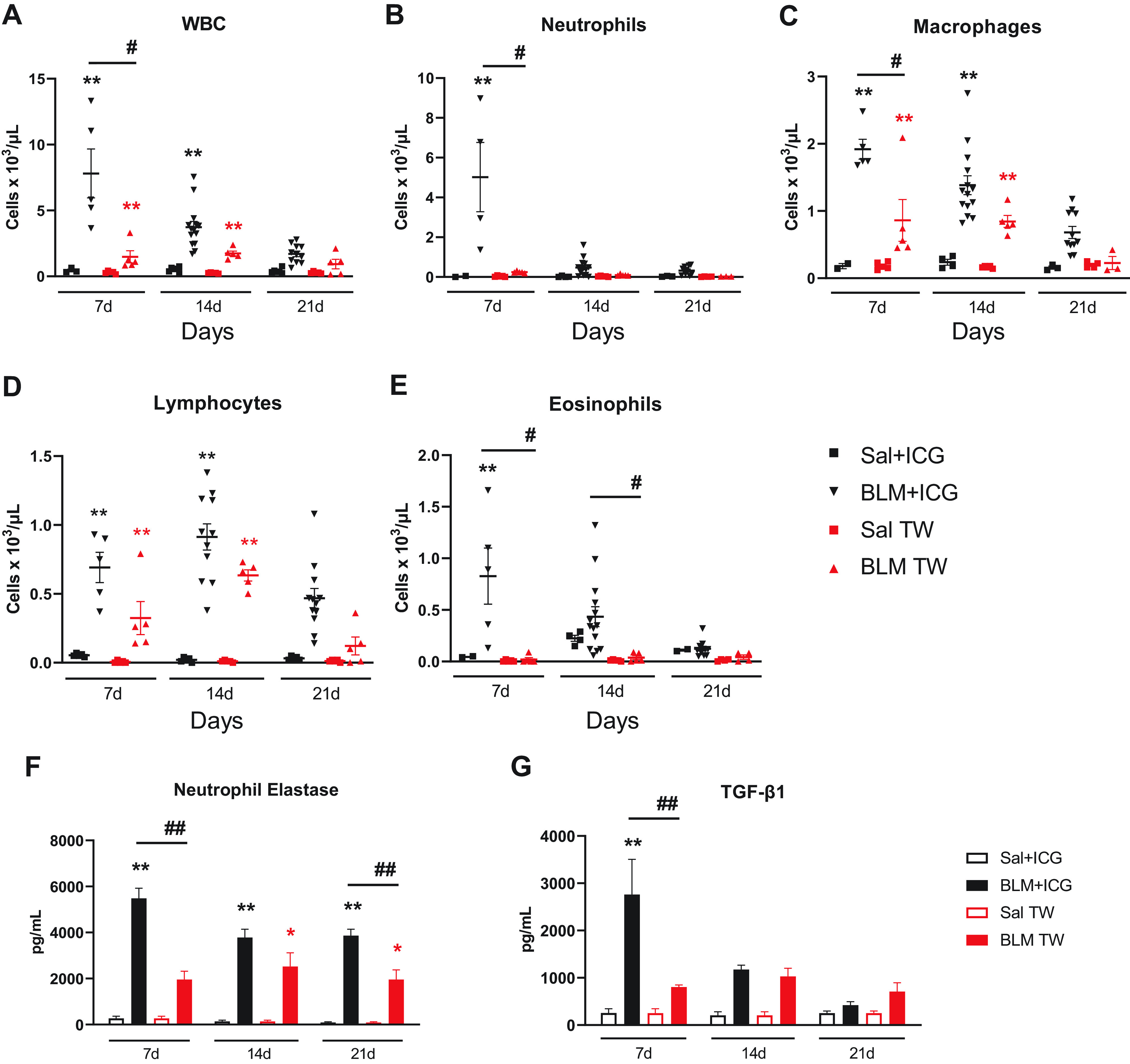
Inflammatory cells profiling, neutrophil elastase, and TGF-β1 levels in the BAL fluid. Leukocyte infiltration of the BALF from differently treated (Sal+ICG, BLM+ICG, Sal, and BLM) groups. Time-dependent variation in the abundance of total white blood cells (WBC; *A*), neutrophils (*B*), macrophages (*C*), lymphocytes (*D*), and eosinophils (*E*). Data are the means ± SE of three independent experiments. Two-way ANOVA with Sidak’s correction for multiple comparisons was applied to the following number of animals (*n*): Sal, Sal+ICG, and BLM groups, *n* = 5 for each time point; BLM+ICG groups, *n* = 12 for each time point; *P < 0.05, **P < 0.01, BLM+ICG vs. Sal+ICG; #*P* < 0.05 BLM+ICG vs. BLM-only group. *F*: neutrophil elastase levels measured in the BAL of Sal+ICG, BLM+ICG, Sal, and BLM mice at *days 7, 14*, and *21*. *G*: TGF-β1 measured in the BAL of Sal+ICG, BLM+ICG, Sal, and BLM mice at *days 7, 14*, and *21*. Data, expressed as pg/ml, are the means ± SE of three independent replicates. Two-way ANOVA with Tukey’s test for multiple comparisons was used to compare treatment/control pairs (BLM+ICG vs. Sal+ICG group and BLM vs. Sal) at each of the indicated time points. Statistical analysis was applied to the following number of animals (*n*): Sal, Sal+ICG, and BLM groups, *n* = 5 for each time point; BLM+ICG groups, *n* = 12 for each time point. **in black *P* < 0.01 BLM+ICG vs. Sal+ICG; *in red *P* < 0.05 BLM vs. Sal; ##*P* < 0.01 BLM+ICG vs. BLM group. BAL, bronchoalveolar lavage; BLM, bleomycin; ICG, indocyanine green; Sal, saline.

As inflammation is an early hallmark of bleomycin-induced fibrosis, we evaluated the development of pulmonary inflammation by measuring the number of inflammatory cells (Fig. 6) and cytokine levels (Supplemental Table S1) in the bronchoalveolar lavage fluids (BALFs) from animals belonging to the various groups (Sal + ICG, Sal, BLM + ICG, and BLM-only treated mice).

Total white blood cells (WBCs), including neutrophils, macrophages, and eosinophils, were all significantly increased at *day 7* in BALFs from BLM + ICG compared with Sal + ICG mice (*P* < 0.01; *P* < 0.01; *P* < 0.01, and *P* < 0.05, respectively), which did not exhibit any appreciable inflammatory response signature at any of the examined time points (Fig. 6, *A*–*E*). This effect decreased at *day 14*, but relatively high levels of total WBCs and macrophages (*P* < 0.01) could still be detected at this time point (Fig. 6, *A* and *C*). A delayed peak of lymphocyte levels (*P* < 0.01) was also apparent at *day 14* (Fig. 6*D*), indicating a switch in the mediators involved in disease maintenance. At *day 21*, all inflammatory cells returned to basal levels, without any significant difference between BLM + ICG-treated mice and the other groups of animals.

As expected, BLM was the second most effective treatment at increasing the levels of all inflammatory cell types except neutrophils and eosinophils at *days 7* and *14* (*P* < 0.01 compared with the Sal and Sal + ICG groups). As in the case of BLM + ICG, inflammatory cells (total WBCs, macrophages, and lymphocytes) in the BLM-only group returned to basal levels at *day 21*, thus pointing once again to the fully reversible nature of the inflammatory response associated with the early phase of BLM-induced lung fibrosis. In addition to the lack of any appreciable effect of the BLM-only treatment on neutrophils and eosinophils, a significantly reduced increase in WBCs and macrophages (*P* < 0.05) was observed in this group, at *day 7*, compared with BLM + ICG-treated animals. A similar, albeit not significant trend was observed for lymphocytes at *days 7* and *14*, thus indicating the induction of a synergistic enhancement of multiple inflammatory cell types by BLM + ICG compared with BLM- and ICG-only treatments. This effect was partly mirrored by a parallel, statistically significant increase, especially at *day 7*, of 7 of the 17 tested cytokines (nine were not detectable) in the BALFs from BLM + ICG compared with control Sal + ICG animals (Supplemental Table S1). Also notable, and in keeping with the emphysema-like phenotype highlighted by micro-CT and histomorphometric analyses, was the striking elevation of elastase levels detected in the BALFs of BLM + ICG- but not Sal + ICG-treated animals (*P* < 0.01; Fig. 6*F*). Importantly, elastase levels were markedly higher in the BLM + ICG group than in mice receiving a double BLM-only treatment via OA administration (*P* < 0.01). Given the presumable neutrophil origin of elastase, which is considered a hallmark of emphysema and is used as an experimental tool to induce this particular lung pathology (35), the observed increase of this enzyme upon treatment with BLM + ICG also fits with the enhanced BALF neutrophil levels that were induced by this compound combination but not by any single-compound treatment (Fig. 6*B*). We also measured TGF-β1 levels in BAL samples from Sal + ICG-, BLM + ICG-, Sal-, and BLM-treated mice (Fig. 6*G*). Even though an increase in TGF-β1 levels was detected in BLM mice compared with the saline control group, these were markedly higher in BLM + ICG- than in BLM-only-treated mice (*P* < 0.01) at *day 7*.

### Proapoptotic and Cell Morphology Alterations Induced by BLM + ICG Ex Vivo and In Vitro

**Figure 7.**
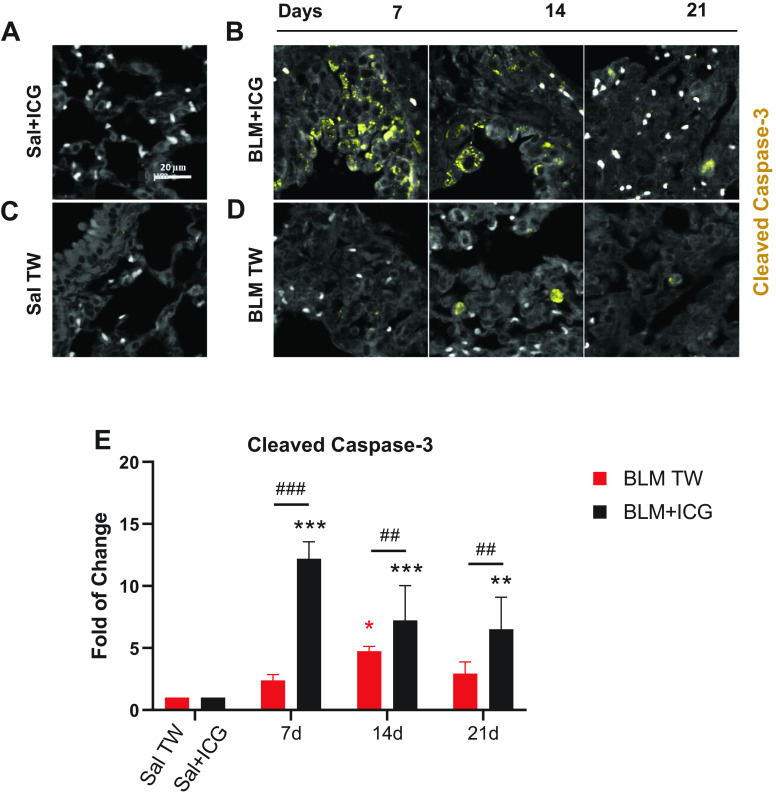
Cleaved Caspase-3 immunofluorescence staining of lung parenchyma. Representative immunofluorescence micrographs of cleaved Caspase-3-stained lung parenchyma (×20 magnification; scale bar 20 µm) from mice treated with Sal+ICG (*A*) and BLM+ICG (*B*) at the indicated times post-OA. White dots represent red blood cells. *C* and *D* are the same as *A* and *B* for the Sal (*C*) and the BLM-only (*D*) groups. For the Sal+ICG and Sal control groups, cleaved Caspase-3 signal remained essentially constant throughout all time points. Cleaved Caspase-3 was stained with TRITC (see materials and methods for details). *E*: immunofluorescence data quantitation and time-dependent variation of cleaved Caspase-3-positive cells in mice treated with BLM+ICG or BLM, using Sal+ICG- and Sal-treated animals as controls. Data are expressed as fold-change relative to the respective control group. Asterisks indicate statistical significance vs. the corresponding control groups (*P < 0.05, **P < 0.01, ***P < 0.001). Hashtags indicate the significance of intergroup (BLM+ICG vs. BLM-only) differences (##*P* < 0.01; ###*P* < 0.001). BLM, bleomycin; ICG, indocyanine green; OA, oropharyngeal aspiration; Sal, saline.

As revealed by in vivo data, the BLM + ICG combination triggered a strong tissue reaction (considerably stronger and more composite than the one induced by BLM alone) ultimately causing lung fibrosis with emphysema-like features after OA administration. To gain initial insight into the molecular bases of this reaction, we investigated the effects of BLM and ICG, both alone and in combination, both in vitro, through cell-based assays, and ex vivo, on paraffin-embedded lung sections. The morphological appearance of apoptosis is shown in Fig. 7, *A–D*. The number of Caspase-3-positive cells was markedly higher in BLM + ICG mice relative to both control (Sal + ICG) and BLM mice at *day 7*. At variance with the BLM-only group, in which active caspase-3 was detected in cell nuclei at *day 14*, apoptotic cells were visible at all time points in the BLM + ICG group, as confirmed by cleaved Caspase-3 signal quantification (Fig. 7*E*). In BLM + ICG mice, the levels of cleaved Caspase-3 were markedly higher as than in Sal + ICG (*P* < 0.01), as well as in BLM mice (*P* < 0.01), at each of the examined time points. On the other hand, as regard the BLM group, a significant increase in cleaved caspase-3 with respect to the Sal control group was detected only at *day 14* (*P* < 0.05), whereas a slightly, not significantly, higher signal was observed at the other time points.

**Figure 8.**
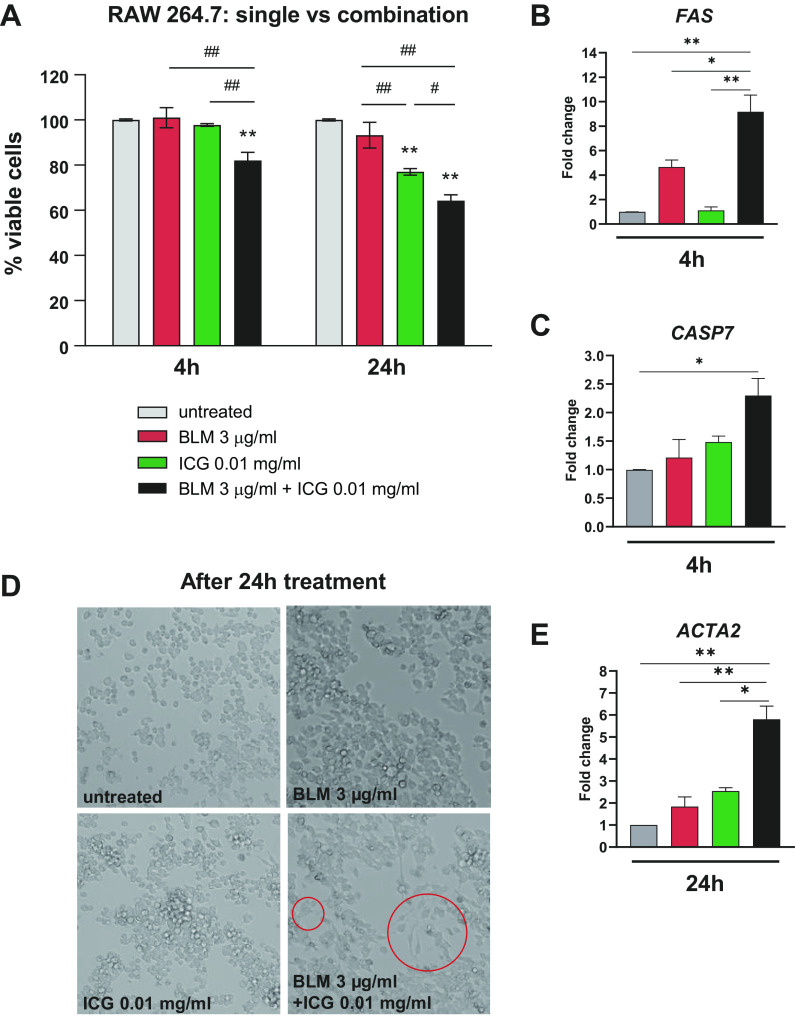
In vitro evaluation of the cellular effects of the combined BLM+ICG treatment. *A*: MTT-based viability assays performed on RAW 264.7 macrophages treated with BLM (3 µg/mL) and ICG (0.01 mg/mL), both as single agents and in combination (the results of similar experiments performed on the murine epithelial cell line LA-4 are shown in Supplemental Fig. S5*A*). Data are the means ± SE of three independent experiments. Two-way ANOVA with Tukey’s test for multiple comparisons was used to statistically evaluate the data: **P < 0.01 is the significance of individual treatments vs. the untreated control condition at each time point; #*P* < 0.05, ##*P* < 0.01 (shown above the bars on a horizontal line) is the significance of comparisons between different treatment groups. *B*: *FAS* mRNA levels determined by RT-PCR in RAW 264.7 cells treated for 4 h with BLM and ICG alone, or in combination; data are expressed as fold-change relative to the untreated (Sal) control. *C*: same as *B* but applied to the *CASP7* mRNA; the results of similar experiments performed on the epithelial cell line LA-4 are shown in Supplemental Fig. S3, *B* and *C*. One-way ANOVA with Tukey’s test for multiple comparisons was used for statistical analysis: *P < 0.05, **P < 0.01. *D*: bright-field micrographs of RAW 264.7 cells treated for 24 h with BLM (3 µg/mL) and ICG (0.01 mg/mL), both as single agents and in combination. Red circles indicate macrophages that have acquired a spindle-like shape after the BLM+ICG treatment. *E*: *ACTA2* mRNA expression levels measured by RT-PCR in RAW 264.7 cells treated with BLM, ICG, and BLM+ICG expressed as fold-change relative to untreated controls (set to 1.0). One-way ANOVA with Tukey’s test for multiple comparisons was used to evaluate statistical significance: *P < 0.05, **P < 0.01. Data are the means ± SE of three independent biological replicates. BLM, bleomycin; ICG, indocyanine green.

In vitro assays were conducted on the murine macrophage cell line RAW 264.7, using previously reported concentrations (30–32) of ICG (0.01 and 0.1 mg/mL) (Supplemental Fig. S5). This cell line was chosen because it represents the major source of profibrotic cytokines in lung fibrosis development and is easy to culture (36). To keep a BLM/ICG molar ratio similar to that used for in vivo experiments, 3 and 50 µg/mL BLM concentrations were tested. At the highest concentration, BLM significantly reduced the viability of RAW 264.7 cells compared with untreated controls at both 4 h and 24 h (*P* < 0.05 and *P* < 0.01, respectively), whereas no appreciable detrimental effect was observed at the lower (3 µg/mL) concentration (Supplemental Fig. S5). ICG, instead, did not affect cell viability at either concentration after 4 h, but caused a dose-dependent reduction of cell viability at both concentrations after 24 h. The lowest concentrations of BLM and ICG (3 µg/mL and 0.01 mg/mL, respectively), which proved to be ineffective alone after a 4 h incubation, were thus selected for subsequent combination studies. As shown in Fig. 8*A*, the combination of both compounds significantly reduced RAW 264.7 cell viability compared with untreated or single-compound-treated cells (*P* < 0.01) at both 4 and 24 h. Also, apparently unique to the combined BLM + ICG treatment was the synergistic activation of the early extrinsic apoptotic pathway, as suggested by the results of a targeted gene expression analysis that revealed a significant upregulation at 4 h of the mRNAs coding for the death receptor Fas (*P* < 0.01; Fig. 8*B*) and the apoptosis-related peptidase Caspase-7 (*P* < 0.05; Fig. 8*C*).

Interestingly, the BLM + ICG combination also induced a morphological transition of RAW 264.7 cells toward a spindle-like shape at 24 h (Fig. 8*D*). This was accompanied by a marked upregulation of the mRNA coding for the myofibroblast formation marker α-smooth muscle actin *ACTA2*, whose levels significantly increased in BLM + ICG-treated compared with untreated cells (*P* < 0.01), as well as compared with cells treated with BLM (*P* < 0.01) or ICG (*P* < 0.05) alone (Fig. 8*E*).

The effect of BLM and ICG and their combination was also tested in the mouse lung epithelial cell line LA-4. As shown in Supplemental Fig. S6*A*, ICG but not BLM alone significantly reduced cell viability at 4 and 24 h (*P* < 0.01 vs. untreated cells). Also with this cell line, however, a much more pronounced loss of viability was induced by the combined BLM + ICG treatment (*P* < 0.01 vs. the ICG-only control; Supplemental Fig. S6*A*). As in the case of RAW 264.7 cells, loss of cell viability was paralleled by a marked, albeit only partially significant, upregulation of the proapoptotic *FAS* and *CASP7* mRNAs, which was only detectable in BLM + ICG-treated cells (Supplemental Fig. S6, *B* and *C*).

Because of their more favorable morphology compared with macrophages, epithelial LA-4 cells also allowed us to investigate the subcellular localization of the fluorescent dye ICG after a 24 h treatment. As shown by the representative fluorescence microscopy images presented in Supplemental Fig. S6*D*, the red fluorescence signal associated with the ICG dye displayed an exclusively cytoplasmic localization both in cells treated with ICG alone as well as with the BLM + ICG combination.

## DISCUSSION

Although our primary goal was to investigate the lung distribution of BLM associated with a fluorescent dye, we unexpectedly found that ICG displays long-lasting lung retention upon OA administration. Moreover, the BLM + ICG combination triggered a severely aggravated form of pulmonary fibrosis which, unlike the fibrotic phenotype commonly observed in the BLM-only model widely used by our and many other laboratories (8, 15, 16), was associated with distinctive hallmarks of an emphysema-like pathology. These results contrast with both the extremely short circulation half-life commonly reported for intravenously administered ICG, as well as with its widely recognized safety and lack of significant side-effects or cytotoxicity, at least under non-irradiation (photodynamic or photothermal therapy) conditions (14).

Long-lasting lung persistency of ICG has similarly never been reported before. However, we documented the presence of ICG, coadministered with BLM, inside epithelial cells and a persistent (up to 21 days) ICG-associated fluorescence signal in the lungs. A possible explanation for these divergent behaviors may reside in the well-known ability of ICG to tightly bind to proteins as well as phospholipids (PLs), with a significant change in its fluorescence (intensity and half-life) properties (37, 38). Given the high PL and protein content of the lung surfactant (39), it is conceivable to imagine that prolonged retention within the lungs is due to interaction with these surfactant components.

We used multiple experimental approaches, both in vivo and in vitro, to comparatively characterize the lung pathology caused by the BLM + ICG and the BLM-only treatments and to begin to elucidate the mechanisms underlying their quantitative and qualitative different effects. Indeed, as revealed by total Ashcroft score and fibrosis severity assessments based on frequency distribution, the fibrotic phenotype induced by the BLM + ICG combination was significantly more severe than that observed upon BLM instillation. Importantly, the fibrosis enhancement elicited by BLM + ICG was always accompanied by the appearance of hyperinflated areas that were uniquely detected in BLM + ICG-treated mice by micro-CT. The latter areas were interpreted as signs of an emphysema-like lung tissue alteration: an interpretation that was corroborated by the results of histomorphometric and mean linear intercept analyses, both of which highlighted a significant increase of the alveolar air spaces within the lung parenchyma. The increase in air space size was particularly striking at 21 days after OA administration of BLM + ICG and led to a significant reduction of parenchymal tissue.

Fibrillar Collagen type I is one of the main types of collagen in the lung parenchyma. Although Collagen I deposition and its fibrillar assembly are well-known hallmarks of BLM-induced lung fibrosis, emphysema is characterized by more profound changes in the composition of the extracellular matrix, which, in an attempt to repair damaged lung tissue, accumulates defective and unstructured collagen fibers leading to an increased tissue stiffness (40). Thus, even though at *day 21* fibroblasts were still activated in BLM + ICG mice, as revealed by FAP-1α quantification, the levels of Collagen I were not significantly increased. Importantly, we showed that in vivo detection of FAP-1α can assess the fibrotic activity of BLM + ICG-mediated injury, as was recently reported for the BLM model (41). Because of the close temporal association between the development of high-grade fibrosis and the appearance of emphysema-like features, we hypothesize that mechanical traction on lung parenchyma, exerted by the accumulated fibrotic tissue, might be responsible for (or at least contribute to) the observed airspace enlargements and increase of hyperinflated areas. In keeping with the early inflammation commonly observed in response to BLM (42), the distinctive parenchymal alterations induced by BLM + ICG were preceded by a massive and much more sustained inflammatory cells’ (particularly, neutrophils, macrophages, and eosinophils) accumulation in the BALF of BLM + ICG- compared with BLM-only-treated animals. This was paralleled by increased levels of different cytokines, including the proinflammatory marker IL-6, which was again more prominent in mice subjected to the combination treatment. In keeping with the absence of any detectable histomorphometric sign of an emphysema-like transition in BLM-only treated mice, no significant increase in neutrophil levels was observed in this group of animals, thus further indicating a unique causal relationship between the combined BLM + ICG treatment and the development of this particular lung tissue alteration.

Eosinophils and the eosinophilic IL-5 and eotaxin cytokines were also selectively elevated in the BALFs of BLM + ICG-treated mice, especially on *days 7* and *14* after instillation. Even though eosinophilia is not considered a major causal factor of emphysema, eosinophils likely contribute to alveolar destruction (43, 44) leading to the distinctive parenchymal alteration hallmarks observed in the BLM + ICG model.

A mixed fibrotic and emphysematous-like phenotype is observed in other respiratory pathologies, most notably chronic obstructive pulmonary disease (COPD), in which neutrophils, but often also eosinophils, are considered the main inflammatory cells (45).

It is thus tempting to speculate that the herein described BLM + ICG model, with its characteristic inflammatory neutrophilic/eosinophilic BALF infiltration profile, and peculiar late-stage lung morphological alterations, may actually share a significant similarity with COPD. This is further corroborated by a marked elevation, especially at *day 7*, of neutrophil-derived elastase, a proteolytic enzyme causally associated with emphysema development and widely used as an experimental tool to induce this particular lung pathology (35). Moreover, Caspase-3, a physiological mediator of apoptosis, has been shown to play an important role in emphysema pathogenesis (46). Interestingly, cleaved Caspase-3 was markedly elevated only in the lungs of BLM + ICG mice, especially at 7 days after OA administration, and this was accompanied by a concomitant increase in TGF-β1, a fibrotic driver in the context of lung injury (47).

In in vitro assays, BLM + ICG significantly decreased the viability of macrophage and epithelial cell lines and upregulated the expression of two mRNAs (*FAS* and *CASP7*) coding for two known drivers of the extrinsic apoptotic pathway. In the case of macrophages, this was paralleled by the upregulation of the mRNA coding for the myofibroblast formation marker α-smooth muscle actin (*ACTA2*) and by the switch from a round to a spindle-like morphology, which has previously been associated with a proinflammatory, rather than wound-healing, phenotype (48). Moreover, macrophage transdifferentiation to collagen-producing myofibroblasts has been reported as one of the key drivers of fibrosis in mice, as well as in humans (49).

Interestingly, even if ICG displayed some toxicity on cell lines, no harmful effect was observed in vivo in mice receiving the dye alone. In fact, despite a well-documented in vitro toxicity (30–32, 50), ICG is widely used for multiple clinical, especially diagnostic, applications (9–11) since many decades, and reported adverse reactions on patients have been extremely rare (51).

Although the detailed mechanism underlying the peculiar synergistic effects of BLM + ICG remains to be determined, the above findings suggest that despite a substantial loss of cell viability, the process(es) triggered by the combined administration of the two compounds cannot be simply ascribed to a generalized, necrotic/cytotoxic effect, but rather to a subtler apoptotic, morphological, and likely function-modifying transition.

### Conclusions

We have described a new, more comprehensive model of lung fibrosis that may represent a useful addition to the currently available toolkit for pulmonary disease drug discovery. As pointed out by the multiple lines of evidence presented in this work, the BLM + ICG combination may allow to overcome a major shortcoming of the commonly used BLM model, namely, the fact that lung fibrosis induced by a BLM-only treatment starts to spontaneously resolve after 14 days, thus hindering the achievement of a proper time-window for testing the efficacy of new drug candidates. In fact, BLM + ICG-induced fibrosis develops early and maintains a high level of severity after 21 days. Although the fibrotic peak occurs at 14 days, the BLM + ICG-induced injury is associated with progressive emphysema-like features that resemble human IPF, as well as certain forms of COPD. A lung fibrosis animal model alternative to bleomycin is strongly needed; in fact, Nintedanib, an approved drug for IPF (52), as well as the PDE4B inhibitor BI-101550 (53), were tested in BLM and silica models, even though the silica model is not fibrosis-specific and is associated with a strong inflammatory response.

From a methodological point of view, the present data also document the importance of functional micro-CT analysis as a crucial exploratory tool for longitudinal, qualitative as well as quantitative in vivo monitoring of disease progression.

We thus strongly believe that a continuous and integrated refinement of both the animal models and the bioanalytical tools (micro-CT, but also histomorphometric analyses) that are applied to pharmacologically oriented studies of lung fibrosis and other pulmonary pathologies are essential prerequisites for a successful preclinical investigation with a high potential for clinical translation.

## ETHICAL APPROVALS

All animal experiments described herein were approved by the intramural animal-welfare committee for animal experimentation of Chiesi Farmaceutici under protocol number: 809/2020-PR and complied with the European Directive 2010/63 UE, Italian D.Lgs 26/2014.

## DATA AVAILABILITY

The data sets used and/or analyzed during the current study are available from the corresponding author on reasonable request.

## SUPPLEMENTAL DATA

10.6084/m9.figshare.20014943 Supplemental Fig. S1: https://doi.org/10.6084/m9.figshare.20014943.

10.6084/m9.figshare.20015039 Supplemental Fig. S2: https://doi.org/10.6084/m9.figshare.20015039.

10.6084/m9.figshare.20015912 Supplemental Fig. S3: https://doi.org/10.6084/m9.figshare.20015912.

10.6084/m9.figshare.21355290 Supplemental Fig. S4: https://doi.org/10.6084/m9.figshare.21355290.

10.6084/m9.figshare.21355368 Supplemental Fig. S5: https://doi.org/10.6084/m9.figshare.21355368.

10.6084/m9.figshare.21776369 Supplemental Fig. S6: https://doi.org/10.6084/m9.figshare.21776369.

10.6084/m9.figshare.20015948 Supplemental Table S1: https://doi.org/10.6084/m9.figshare.20015948.

## GRANTS

This study was fully supported by Chiesi Farmaceutici S.p.A.

## DISCLOSURES

This study was fully supported by Chiesi Farmaceutici S.p.A. F.F.S., G.V., and A.G. are employees of Chiesi Farmaceutici S.p.A., which supported the research work. None of the other authors has any conflicts of interest, financial or otherwise, to disclose.

## AUTHOR CONTRIBUTIONS

A.G., E.F., and F.F.S. conceived and designed research; A.G., E.F., and Z.K. performed experiments; L.M., R.C., M.Z., and L.L. analyzed data; A.G., A.K., C.W.G.M.L., G.D., and F.F.S. interpreted results of experiments; E.F., L.M., R.C., M.Z., L.L., and Z.K. prepared figures; A.G., G.V., and F.F.S. drafted manuscript; A.K., C.W.G.M.L., G.D., and G.V. edited and revised manuscript; A.G., E.F., L.M., R.C., M.Z., L.L., Z.K., A.K., C.W.G.M.L., G.D., G.V., and F.F.S. approved final version of manuscript.
